# Pragmatic clinical trials embedded in healthcare systems: generalizable lessons from the NIH Collaboratory

**DOI:** 10.1186/s12874-017-0420-7

**Published:** 2017-09-18

**Authors:** Kevin P. Weinfurt, Adrian F. Hernandez, Gloria D. Coronado, Lynn L. DeBar, Laura M. Dember, Beverly B. Green, Patrick J. Heagerty, Susan S. Huang, Kathryn T. James, Jeffrey G. Jarvik, Eric B. Larson, Vincent Mor, Richard Platt, Gary E. Rosenthal, Edward J. Septimus, Gregory E. Simon, Karen L. Staman, Jeremy Sugarman, Miguel Vazquez, Douglas Zatzick, Lesley H. Curtis

**Affiliations:** 1 Department of Population Health Sciences, Duke University School of Medicine, 220 W Main St., Suite 720A, Durham, NC 27705 USA; 2 Duke Clinical Research Institute, 2400 Pratt St., Durham, NC 27710 USA; 3 Department of Psychology and Neuroscience, Duke Clinical Research Institute, Durham, NC 27710 USA; 4 Duke University School of Medicine, 3115 N. Duke Street, Durham, NC 27704 USA; 5 Kaiser Permanente Center for Health Research, 3800 N. Interstate Avenue, Portland, OR 97227-1098 USA; 6 Perelman School of MedicineBlockley Hall, Office 920, 423 Guardian Drive, Philadelphia, PA 19104 USA; 7 Kaiser Permanente Washington Health Research Institute, 1730 Minor Ave, Suite 1600, Seattle, WA 98101-1466 USA; 8 University of Washington, 325 Ninth Ave, Box 359728, Seattle, WA 98104-2499 USA; 9 University of California Irvine School of Medicine, 101 The City Drive South, City Tower, Suite 400, Mail Code: 4081, Orange, CA 92868 USA; 10 Group Health Research Institute, 1730 Minor Ave, Suite 1600, Seattle, WA 98101-1466 USA; 11 Department of Community Health, Brown University, Box G-S121-2, Providence, RI 02912 USA; 12 Department of Population Medicine, Harvard Pilgrim Health Care Institute, 401 Park Drive, Suite 401 East, Boston, MA 02215 USA; 13 Department of Internal Medicine, Wake Forest School of Medicine, Medical Center Boulevard, Winston-Salem, NC 27157 USA; 14 Hospital Corporation of America Nashville TN, AND Texas A&M College of Medicine, One Park Plaza, Nashville, TN 37203 USA; 15 CHB Wordsmith, Inc., 22 Bagwell Ave, Raleigh, NC 27607 USA; 16 Johns Hopkins Berman Institute of Bioethics, 1809 Ashland Ave., Room 203, Baltimore, MD 21205 USA; 17 University of Texas Southwestern Medical Center, 5323 Harry Hines Boulevard, Dallas, TX 75390-8856 USA; 18 University of Washington School of Medicine, 325 9th Ave, Seattle, WA 98104 USA

**Keywords:** Embedded clinical trials, Pragmatic research, Pragmatic clinical research, Cluster randomized trials, Stakeholder engagement

## Abstract

**Background:**

The clinical research enterprise is not producing the evidence decision makers arguably need in a timely and cost effective manner; research currently involves the use of labor-intensive parallel systems that are separate from clinical care. The emergence of pragmatic clinical trials (PCTs) poses a possible solution: these large-scale trials are embedded within routine clinical care and often involve cluster randomization of hospitals, clinics, primary care providers, etc. Interventions can be implemented by health system personnel through usual communication channels and quality improvement infrastructure, and data collected as part of routine clinical care. However, experience with these trials is nascent and best practices regarding design operational, analytic, and reporting methodologies are undeveloped.

**Methods:**

To strengthen the national capacity to implement cost-effective, large-scale PCTs, the Common Fund of the National Institutes of Health created the Health Care Systems Research Collaboratory (Collaboratory) to support the design, execution, and dissemination of a series of demonstration projects using a pragmatic research design.

**Results:**

In this article, we will describe the Collaboratory, highlight some of the challenges encountered and solutions developed thus far, and discuss remaining barriers and opportunities for large-scale evidence generation using PCTs.

**Conclusion:**

A planning phase is critical, and even with careful planning, new challenges arise during execution; comparisons between arms can be complicated by unanticipated changes. Early and ongoing engagement with both health care system leaders and front-line clinicians is critical for success. There is also marked uncertainty when applying existing ethical and regulatory frameworks to PCTS, and using existing electronic health records for data capture adds complexity.

## Background

High-quality evidence regarding the risks and benefits of treatments is lacking across a multitude of clinical specialties [1–7], and the clinical research enterprise is not focused on generating the kind of information that drives national guidelines for clinical care. Similarly, traditional clinical trials are often unable to provide high quality evidence in a timely or cost-effective manner [8, 9]. High quality evidence is typically generated by conducting randomized controlled trials (RCTs) using costly stand-alone, non-reusable systems, which may be separate from clinical care, designed with rigorous inclusion and exclusion criteria, and conducted under ideal conditions [10]. These design features are a double-edged sword: they help ensure that findings reflect true variation as a result of an intervention, but have been criticized as being so specific as to not be generalizable to broader populations and settings. Many are calling for a change, including the National Academies of Medicine (formerly the Institute of Medicine), to a system in which data for research are gathered during routine clinical care to enable continual learning, i.e., a “learning healthcare system” [11].

Pragmatic clinical trials (PCTs) are trials that use data collected in the electronic health record (EHR) as part of routine care, or are “embedded” in routine care, and are a foundational component of such a system. By their nature, PCTs are designed to show real-word effectiveness in broad, generalizable patient groups (as opposed to the more restricted protocols and populations found in exploratory randomized trials) [12]. These PCTs have the potential to significantly decrease the evidence gap and inform real-world practice with digital health data collected at the point of care. They involve critical partnerships between health care systems and academic investigators to embed clinically meaningful research questions into the infrastructure of the health system to generate real-world generalizable results in an efficient manner.

While embedded PCTs hold great promise and much work has been done to describe the continuum of pragmatic versus explanatory trials for considerations in the design phase of PCTs [13, 14], information summarizing real-world experiences and best practices with PCTs is scant; there is no clear framework for deciding when PCTs would be optimal. The current ethical, regulatory and logistical systems were created primarily with more “traditional” RCTs in mind [15], and policy makers are still working to understand the unique challenges associated with PCTs. To speed this learning process, the Common Fund of the National Institutes of Health (NIH) created the Health Care Systems Research Collaboratory (Collaboratory) in 2012. The Collaboratory’s mission is to strengthen the national capacity to implement cost-effective, large-scale PCTs, by supporting the design and execution of a series of pragmatic trials, or demonstration projects (Table 1), with the intent to learn how best to design, conduct, and disseminate the results of these PCTs. To date, the Collaboratory has funded the ten demonstration projects described here and worked with the Project Principal Investigators (PIs) to overcome challenges and barriers to designing, conducting, and disseminating results from their PCTs. In addition, an NIH funding opportunity announcement (RFA-RM-16-019) was developed as a part of the Common Fund initiative to support more projects over the next several years. These projects address questions of public health importance, include a large, generalizable population of patients, and engage healthcare delivery organizations as research partners [16]. Members of the Collaboratory Core Groups (described in more detail below) worked with the PIs to facilitate the PCTs and have reported on solutions to the challenges encountered. These include a special issue on the ethics of research in usual care settings [17, 18], a special issue on the ethical and regulatory complexities of pragmatic clinical trials [15, 19–29], journal articles on initiating and implementing patient-reported outcomes measures [30], electronic health records, phenotyping, and informatics [31–33], stakeholder engagement and health care systems interactions [34–37], as well as biostatistical lessons learned on cluster and constrained randomization [38, 39]. Members of the Collaboratory have also developed a Living Textbook on the design, conduct, and dissemination of PCTs (http://rethinkingclinicaltrials.org/).

**Table 1:** NIH Collaboratory Demonstration Projects

Study name	Project goal	Population	Randomization
2012			
Pain Program for Active Coping and Training (PPACT; NCT02113592)	Help patients adopt self-management skills for chronic pain, limit use of opioid medications, and identify factors amenable to treatment in the primary care setting [2]	Patients with chronic pain on long-term opioid therapy (~1000+ patients) in three staff model health plans	Cluster randomization by primary care provider
Strategies and Opportunities to Stop Colorectal Cancer in Priority Populations (STOP CRC; NCT01742065)	Improve the rates of colorectal-cancer screening by mailing fecal immunochemical testing tests to patients at Federally Qualified Health Centers	Individuals eligible for screening per the US Preventive Task Force guidelines (~35,000 patients) in 26 Federally Qualified Health Center clinics	Cluster randomization by Federally Qualified Health Center clinic
Suicide Prevention Outreach Trial (SPOT; NCT02326883)	Compare outcomes in patients who receive care-management or online skills training for suicide prevention versus usual care in three healthcare systems	Individuals at elevated risk for suicide on a depressions scale (~19,500 patients) in three large healthcare systems	Individual randomization
Time to Reduce Mortality in End-Stage Renal Disease (TiME; NCT02019225)	Determine whether increasing the durations of hemodialysis sessions reduces mortality and hospitalization rates for patients receiving maintenance hemodialysis care	Adults who have initiated treatment with maintenance hemodialysis within the past 120 days (~6800 patients) in 266 dialysis facilities operated by two dialysis provider organizations	Cluster randomization by dialysis facility
Lumbar Image Reporting with Epidemiology (LIRE; NCT02015455)	Determine if inserting epidemiological benchmarks (essentially representing the normal range) into lumbar spine imaging reports reduces subsequent spine-related tests and treatments	100 clinics (~230,000 patients) in four health systems	Cluster randomization by clinic. Stepped-wedge one-way crossover design
Active Bathing to Eliminate Infection (ABATE; NCT02063867)	Determine if using antiseptic bathing for all patients plus nasal ointments for patients harboring methicillin-resistant *Staphylococcus aureus* (MRSA) reduces multidrug-resistant organisms and bloodstream infections	Patients in adult medical, surgical, oncology, and step-down units. (~600,000 patients) in 53 hospitals	Cluster randomization by hospital
Blood Pressure Medication Timing Study (BPMedTime)	To compare adverse cardiovascular events in patients who are instructed to take their currently prescribed once-daily antihypertensive medications at bedtime compared with patients who continue to take their once-daily antihypertensive medications in the morning or afternoon	Initial plan was to test this question in 1000 patients, but to have enough power, it was determined that 5000 patients were needed	This trial was not transitioned to the implementation phase
2014			
Pragmatic Trial of Video Education in Nursing Homes (PROVEN; NCT02612688)	Determine if showing advance care planning videos in nursing homes affects the rates of hospitalization among frail, demented residents	2 nursing home health systems (360 nursing homes) serving long-stay (>12 months) patients with advanced comorbid conditions (~15,000 patients)	Cluster randomization by nursing home
Improving Chronic Disease management with Pieces (ICD-Pieces; NCT02587936)	Improve care for patients with chronic kidney disease, diabetes, and hypertension by using a novel technology platform (Pieces) that uses the electronic health record to identify patients and by assigning practice facilitators within primary care practices or community medical homes	Patients with multiple co-morbid conditions (chronic kidney disease, diabetes, and hypertension; ~11,000) in 124 primary care practices in four healthcare systems	Stratified cluster randomization of clinical practices within health systems
Trauma Survivors Outcomes and Support (TSOS; NCT01625416)	Coordinate care and improve outcomes for trauma survivors with post-traumatic stress disorder(PTSD) and comorbidity	Expect 40 patients at each center (~960 patients) in 24 US trauma centers	Cluster randomization by trauma center. Stepped-wedge one-way crossover design

This table is based on a table previously published by the Collaboratory in a white paper titled *Lessons Learned from the NIH Health Care Systems Research Collaboratory Demonstration Projects* and is used with permission [41].

Although there have been numerous publications describing nuanced challenges and solutions encountered in the conduct of specific Demonstration Projects, this is the first to widen the focus and describe the NIH Collaboratory more generally. We build on the knowledge created by the Cores to review important generalizable lessons learned over the first four years since the Collaboratory was established. We also discuss the remaining opportunities and challenges for the Collaboratory and its forthcoming projects, and for the medical community more broadly regarding PCTs to power a learning health system.

## Rationale and organization of the NIH Collaboratory

To provide expertise to the NIH Collaboratory Demonstration Projects, the following working groups, or Cores were created to explore key elements of PCTs: electronic health records; phenotypes, data standards and data quality; patient-reported outcomes; health care systems interactions; ethics & regulatory; biostatistics and study design; and stakeholder engagement. The Collaboratory Coordinating Center  at the Duke Clinical Research Institute (with colleagues at the Johns Hopkins Berman Institute of Bioethics, Harvard Pilgrim Health Care Institute, Group Health Research Institute/Kaiser Permanente Washington Health Research Institute, Center for Medical Technology Policy, and Medical College of Wisconsin) serves 3 roles: (1) to provide support to the demonstration projects to help ensure success; (2) to cull experiences and create generalizable solutions regarding challenges in designing, conducting, and disseminating results from PCTs; and (3) to disseminate lessons learned, tools, and other resources to the broader community, including the creation of a Knowledge Repository and the Living Textbook of Pragmatic Clinical Trials: *Rethinking Clinical Trials*.

## Collaboratory demonstration projects

There have been two funding opportunities so far, one in 2012 and one in 2014, and we expect another funding opportunity in 2017 to support 10 additional PCTs. In 2012, seven demonstration projects were funded (RFA-RM-12-002, Table 1), and in 2014, three additional demonstration projects were added to the Collaboratory to focus on interventions that improve the health of patients with multiple chronic conditions (RFA-RM-13-012). In recognition of the challenge of conducting trials in ongoing clinical care settings and other uncertainties associated with their design, a two-phase, cooperative agreement approach was used for their funding. In the one-year planning phase, the demonstration project investigators refined their study protocols in cooperation with other experts within the Collaboratory network and Core groups, piloted aspects of the proposed designs, and developed revised proposals for their projects that were submitted to NIH. Projects deemed acceptable were funded for the four-year execution phase. This two-phase approach was beneficial in that it (1) drew on the broader intellectual community of the Collaboratory to contribute to the planning of each project, (2) highlighted the questions and uncertainties that arise during the planning of PCTs within healthcare systems, and (3) increased the likelihood that the projects would be successful in answering their scientific question.

## A Spirit of openness

Because the Collaboratory pragmatic trial interventions are tested in the context of routine medical care, data are collected from the EHR and claims, and the studies involve large sample sizes, the engagement of healthcare delivery organizations, providers, researchers, and representatives from the NIH (12 NIH institutes and centers are involved) has been critical. The Collaboratory’s success depends entirely on the willingness of investigators to share their challenges in real time with the rest of the network, including the NIH staff. This differs from many research enterprises, in which investigators often feel motivated to present an image of perfection to everyone outside of their project and especially to the sponsors. The Collaboratory projects were selected with the understanding that they would generate challenges that the research community must learn to overcome. The transformation of challenges into lessons learned that are then shared with the broader community is a direct result of the good faith, generosity, and candor of the Collaboratory demonstration project investigators and their health system partners.

## Methods

The information regarding lessons learned was gleaned from a number of sources. The Collaboratory held yearly Steering Committee meetings (Bethesda, MD, 2012–2107) in which all the Principal Investigators (PIs) gather to share their experience and knowledge through interviews and presentations. Members of the Coordinating Center interview each of the PIs at these meetings to gather lessons learned, and each PI presents lessons learned at a general session followed by an opportunity for discussion. Leaders of each of the Cores also hold monthly calls with the PIs and their teams to discuss problems and explore solutions, and these lessons are gathered as a part of minutes from the call. In addition, all the PIs and some members of their teams are authors on this paper.

## Results: Lessons learned

### A planning phase is essential

As one of the Collaboratory investigators said, nothing is “plug and play” in a PCT within a healthcare system. In all the Collaboratory demonstration projects, there were important changes made to the protocol during the planning phase as a result of further engagement with stakeholders, consultation with other experts, and collection of pilot data.

In one of the first seven planning projects funded—the Blood Pressure Medication Timing Study (BPMedTime)—the ultimate sample size needed was determined to be 5000 versus the original 1000 subjects in order to detect a lower effect rate than anticipated. Because this new sample size was too large to be feasible within the budgetary constraints of the funding mechanism, the BPMedTime trial was not transitioned to the implementation phase; however, all of the other demonstration projects were moved forward.

### Despite good planning, new challenges arise during execution

Because PCTs occur in the dynamic environment of one or more healthcare systems, changes can arise that require modification of the protocols or how they are implemented. Leadership changes, staff turnover, and new local or national policies can all require a change of plan. For example, after experiencing issues with study implementation, the STOP CRC research team partnered with practice improvement facilitators who were trained in the plan-do-study-act (PDSA) method [40]. The facilitators held in-person meetings with leadership teams from all sites and asked the sites to submit a PDSA plan for issues with the trial. For example, when there were too many fecal kits submitted without a collection date, the “Plan” was to test new patient materials that prompted patients to write the collection date on the kits. PDSA cycles empowered clinics to identify and address local problems and provided information about implementation challenges, which improved study conduct [41]. Additionally, the PROVEN trial had excellent experience integrating a new record documenting the video being offered and shown in nursing homes’ EMR during the pilot implementation, but this failed to reveal that staff were keen to document that the videos were offered even though far fewer patients were actually viewing them. Mid-course corrections required a new approach in which nursing home leadership became even more involved in advocating for the goal of personalized advance care planning.

### Early and ongoing engagement with the healthcare system is critical

Collaboratory PCTs are conducted in real-world settings and leverage the existing infrastructure (e.g., data systems) of healthcare systems to answer clinical questions of importance to patients, providers, payers, and other stakeholders. In these PCTs, the data originate from the healthcare systems EHR and include proprietary information, so strong and trusting partnerships are needed between investigators and healthcare system leaders [35]. While healthcare systems should be committed to improving care for all their patients, they have operational priorities, finite financial resources and institutional energy, and the physicians, leaders and staff in most systems are already pressed for time attending to patient needs. Early engagement with leaders of healthcare systems will facilitate systems identifying partners to help align research goals with organizational goals and performance improvement initiatives [10]. Assistance at the level of the individual provider group is often necessary to identify the best way to incorporate a new intervention into standard-of-care operating procedures.

### Early and ongoing engagement with clinicians is critical

Data are collected during the course of routine care, and partnerships must be built with healthcare providers to ensure that the intervention can be added into existing workflows as seamlessly and with as little burden as possible [42]. Physicians, nurses, and other clinical staff who will implement the protocol will have insight about the feasibility and sustainability of the intervention [43]. For example, the PPACT trial has an intervention that is delivered in the primary care setting where schedules are busy and space is tight. The research team partnered with clinicians to understand the clinical workflow and concerns of integrating research. As a result, they scheduled study-related patient visits during slower clinic periods and held patient visits in less conventional ways, such as after hours and by having groups meet in lobby spaces [41]. The TiME trial investigators found that even small changes to work flow, such as incorporating methods for documenting trial activities, were viewed as large changes by facility staff, and required more effort at the local level than had been anticipated. The PROVEN trial initially asked facility admission staff to incorporate offering and showing the video into the standard admission process, but found that offering this too early was challenging because the existing admission process is already intense. Consequently, the team altered the limits on when the video should be offered. As another example, the investigative team with ICD-Pieces initially planned for structured, step-wise electronic tools that were time consuming but that would provide a detailed therapy plan for patients; after discussing the tool with medical directors and physicians, they developed more user-friendly, less burdensome tools [41]. The clinical decision support tools were streamlined to rely on more user-friendly links to simple workflows, targeted protocols, and related order sets.

### Uncertainty arises when applying existing ethics and regulatory frameworks to embedded pragmatic clinical trials

High quality PCTs should provide meaningful answers to important clinical questions while protecting the rights and welfare of all research participants (which may include clinicians in the case of cluster-randomized studies) and complying with relevant policies and regulations [44, 45]. As the Collaboratory trials were being planned, discussions among investigators and institutional and federal officials involved in human subjects oversight highlighted several areas of ambiguity and, sometimes, disagreement. These issues are reviewed in a series of Collaboratory publications [15, 19–29, 46]. For example, Institutional Review Board process for the SPOT trial took 10 months longer than was typical for large trials at the host institution. The SPOT trial tests suicide prevention strategies versus usual care in patients at risk for suicide, and a fundamental issue arose regarding whether one could conduct a minimal-risk study in a high-risk population [47]. The TiME trial, which also enrolls a high-risk population (patients with end-stage renal disease), was faced with the question of whether a trial with an outcome of mortality could be considered “minimal risk” [27]. For both studies, it was ultimately decided that the incremental risk of the research could be considered minimal, in part because both physicians and patients maintained autonomy with respect to implementation of the intervention.

As another example, the data safety monitoring board (DSMB) overseeing the TSOS trial initially wanted to require the team to report every hospitalization as a serious adverse event, but in their cohort of people at risk for post-traumatic stress disorder (PTSD), approximately 15–20% were expected to be re-hospitalized for non-emergent reasons, and some negotiation was required [41]. In the end, it was decided that only emergency hospitalizations (e.g., hospitalization after a suicide attempt) would be considered serious adverse events that required additional reporting.

There is clearly an opportunity for more learning and reflection regarding the best way to promote the rights and welfare of research participants in the context of PCTs.

## Comparisons between arms can be complicated by unanticipated changes in either arm

In a traditional randomized, controlled trial, it is possible to exert significant control over what happens in the different arms of the trial. Because the demonstration projects are tackling issues that are major public health concerns, such as opioid prescribing and hospital-based infections, competing initiatives are likely to be launched at some of the study sites. Conversely, providers in the experimental arm might resist the intervention due to burden. Providers in the “usual care” arm might voluntarily adopt the trial intervention, especially if there is growing evidence that the intervention is associated with better outcomes. There were a number of opioid reduction related initiatives rolled out during the implementation of PPACT, thereby changing the nature of the underlying usual care. In the words of Lynn DeBar, PI of the PPACT trial: “Our control is not controlled. We can’t control it, and that’s a big challenge [48].” For another example, because observational data suggest that longer hemodialysis sessions are beneficial, dialysis units in the TiME trial, including some of those randomized to usual care, have increased session durations for their patients [41]. Staff turnover might result in interventions being implemented in different ways over time and across sites. As an example, in the PROVEN trial, some participating nursing homes experienced multiple changes in the advance care planning champion in the first year of the study, requiring retraining and interruptions in patients and family members being offered the videos. It is not surprising, therefore, that facilities with more champion turnover had lower rates of showing the video, particularly to long-stay residents. For another example, in the PPACT trial competing local initiatives requiring highly skilled nurses or behavioral specialists resulted in some instability in the intervention staffing and, consequently, some variability in disciplines represented on the core interdisciplinary intervention team across performance sites over time. These and other factors make it necessary to carefully monitor the activities in all of the sites involved in the study, so that at the very least it will be possible to describe the nature of these changes that might affect the difference between the arms.

## Leveraging existing tools and electronic health record systems adds complexity

Reliance on the EHR for data to support research investigations is challenging for many reasons. First, even when sites are a part of a single corporate entity, local coding varies, and cross-site data standardization is essential. Because no two electronic health record (EHR) systems are alike, the solution requires engagement of local data experts. Tools, such as the ones developed for SPOT do not transfer from one site to another, and local adaptation is necessary. Each health system may have a distinct process or pathway for implementation of an intervention, and a multi-site trial will need to adapt to those. In the case of the SPOT trial, even though all of the sites use Epic, they have different preferred methods for implementing standardized assessment tools, and the investigators needed to adapt to those different preferred pathways.

Additionally, because the EHR is optimized for clinical practice and sometimes billing, integrating study-related data elements into the EHR has implications for clinical workflow and compliance. A seemingly small change to the work flow to accommodate a research project likely could have major implications for the IT system. The investigators in LIRE, a study that adds epidemiological benchmarks to lumbar spine imaging reports, found that even minor modifications to reports met with initial resistance since these required allocations of scarce IT resources. Overcoming this sort of hurdle required close coordination with site PIs and local leadership buy-in. Some cases offer a different scenario: for PROVEN, the two corporate partners use a different EHR but with the help of devoted efforts from corporate leadership, were able to add a new record into their EHR system to permit staff to document each time the video was offered and subsequently shown to the patient or family members.

## Discussion: Next steps and remaining opportunities

### What Else do we need to learn about conducting successful PCTs?

As the first round of demonstration projects approach completion, the Collaboratory will continue to identify challenges and lessons learned regarding health care systems interactions, data analysis, interpretation, reporting, and dissemination to inform the next set of demonstration projects and PCTs in general. These lessons will inform other national evidence generation efforts that are underway, such as the Patient-Centered Outcomes Research Network (PCORnet). To understand the full potential of PCTs to advance healthcare, additional knowledge is needed about research designs that were not a part of the first phase of Collaboratory, but may be a part of the second phase:

#### A vs. B comparisons of treatments for individual patients

While the 9 Collaboratory trials test a variety of interventions, none involve simple comparisons between two or more alternative medical products or treatments for individual patients. There is an ongoing need to know the comparative effectiveness of currently used individual therapies and the benefits and risks of medical products, and to understand how PCTs might help address these needs. For example, trials that conduct head-to-head comparisons between different classes of drugs for the same condition—e.g., first line treatment for hypertension [49]—could raise unique logistic, ethical, and regulatory challenges that would be important to understand and address.

#### Investigative teams with varying experience

The Collaboratory projects all benefitted from research teams that were highly experienced in health services research and clinical trials, and included national leaders in their fields who had deep experience in integration of research into health care delivery. Many of the teams also had experience with their health system partners, and having situation-specific experience was critically important. For example, this experience helped the LIRE investigators understand EHR idiosyncrasies and anticipate challenges, to build upon already established trust, and to leverage previously collected data for pilot purposes. The number of investigators with such backgrounds is small relative to the large number of clinical problems that might benefit from well-designed embedded PCTs. Therefore, it is important to understand what, if any, additional challenges might arise when teams with less experience and expertise, or perhaps more importantly, less familiarity with the host delivery system, attempt to design and conduct a PCT. Experience with this broader population of researcher teams would be useful.

### Education and training in PCTs

Because there are few research teams with the knowledge and experience to conduct successful PCTs, it is critical that experiences and best practices from the Collaboratory and other sources (e.g., PCORnet) are translated into education and training to create a larger pool of competent research teams. Teaching people how to conduct PCTs within healthcare systems will require an understanding of how to partner with health care systems and the rapidly changing world of clinical care; the success of a trial depends upon interactions and negotiations with the leaders of healthcare systems of the sort that cannot be taught in a classroom. Thoughtful approaches are needed to develop experience-based learning in the context of existing networks of PCT trialists and to incentivize the few existing experts to devote time to teaching and mentoring.

### Need for core resources for new PCTs

Until we have learned how best to conduct PCTs in different settings and enough research teams have been trained in how to do so, new PCTs could benefit from a core resource to assist in planning and conducting the trials. In the Collaboratory, the demonstration projects benefitted from core expertise within the Coordinating Center during the planning phase. Challenges continued to surface in some of the trials during the implementation phase, underscoring the importance of having a dedicated core resource available throughout the conduct of a PCT. Such a central resource could also be useful for accumulating the knowledge and experience gleaned across multiple PCTs in a wide range of settings, possibly leading to more efficient and informative PCTs in the future.

### Changing culture and incentive structures to make PCTs easier to do

A number of barriers to successful PCTs are associated with how incentives align with health systems, clinicians, and potential sponsors. As healthcare systems become more complex and larger, there are increasing demands to ensure productivity is maintained for the system at large and for clinicians, and it may be more difficult to achieve alignment and to balance clinical obligations with the added work of PCTs. In particular, new mandates on clinician time, such as using EHRs, make it more difficult to add new activities unless it is within the workflow. There is often no clear framework for rewarding or reimbursing clinicians to enable PCTs, especially if time is needed for an intervention that is not directly reimbursed or rewarded. Finally, it may be difficult for clinicians or health systems to envision the direct impact a PCT may have on their practice.

In order to make PCTs the norm rather than the exception, cultural changes and incentives are needed. Institutional leadership could make developing, participating in, and leading PCTs part of the strategic planning, which generally emphasizes the importance for professional growth and alignment with health system values. These approaches have been used in the setting of healthcare reform as payments shifted from volume-based care to value-based care, demonstrating behavior changes in both the health systems as well as the clinicians supporting the systems.

## Conclusion

The NIH Collaboratory is an important part of the overall effort to improve the national capacity to generate evidence to inform healthcare decisions by patients, providers, and payers. Even before the demonstration projects have completed their work, much has been learned. Planning activities are substantial and should be supported as part of a phased approach, such as the planning and implementation phases used by NIH for the Collaboratory. New challenges frequently arise during execution; change is the only constant, and because of this, early and ongoing engagement with all stakeholders is critical. As the new demonstration projects begin, we hope to learn from these lessons and generate others, as there remain opportunities for additional learning in order to make intelligent investments in PCTs. There is also the need to train a new clinical trials workforce, to train reviewers for PCTs, and align interests for all stakeholders to contribute to a national evidence generation system.
